# Mr. William Mitchell, M.B., C.M. Edin., on the Use of Formalin in the Treatment and Removal of Inoperable Malignant Growth

**Published:** 1899-09

**Authors:** 


Mr. William Mitchell, M.B., C.M. Edin., in an interesting communication to a contemporary on the use of formalin in the treatment and removal of inoperable malignant growth, sums up the points in its favor as follows: 1. It is simple in the extreme, requiring no special apparatus, and can be applied without an anæsthetic. 2. It produces no shock. 3. It does not, like electrolysis, set up a diffuse suppurative process, being not only aseptic, but powerfully antiseptic. 4. It is bloodless, and can be applied to very vascular growths, as this case shows. 5. It has very much greater penetrating power, and hence effects a more rapid removal than the usual escharotics, and its application does not like those give rise to a disintegrative or caustic process, with the resulting discharge, but is what I might term a necropoietic process, and with no discharge whatever. 6. As there is no discharge, scarcely any dressing material is required, and an economy is thus effected. 7. During the paring away of the necrosed parts the microscopic limits of such a tumor can be easily seen on the dry, clean-cut surfaces, and an indication is thus given as to the direction in which it is necessary to proceed further. The pieces removed can be subjected to microscopic examination for the same purpose. 8. Above all the process appears to be efficient and safe if care is taken. The drawbacks are: (1) The pain, which is at times pretty severe but can of course be relieved by an anodyne; and (2) the œdema, which is always annoying, and might if extending to the glottis be fatal.

